# Features of Nodules in Explants of Children Undergoing Liver Transplantation for Biliary Atresia

**DOI:** 10.3390/jcm11061578

**Published:** 2022-03-13

**Authors:** Ana M. Calinescu, Anne-Laure Rougemont, Mehrak Anooshiravani, Nathalie M. Rock, Valerie A. McLin, Barbara E. Wildhaber

**Affiliations:** 1 Swiss Pediatric Liver Center, Geneva University Hospitals, 1205 Geneva, Switzerland; anne-laure.rougemont@hcuge.ch (A.-L.R.); mehrak.dumont@hcuge.ch (M.A.); nathalie.rock@hcuge.ch (N.M.R.); valerie.mclin@hcuge.ch (V.A.M.); barbara.wildhaber@hcuge.ch (B.E.W.); 2 Division of Child and Adolescent Surgery, Department of Pediatrics, Gynecology, and Obstetrics, Geneva University Hospitals, University of Geneva, 1205 Geneva, Switzerland; 3 Division of Clinical Pathology, Diagnostic Department, Geneva University Hospitals, University of Geneva, 1205 Geneva, Switzerland; 4 Unit of Pediatric Radiology, Diagnostic Department, Geneva University Hospitals, University of Geneva, 1205 Geneva, Switzerland; 5 Gastroenterology, Hepatology and Nutrition Unit, Division of Pediatric Specialties, Department of Pediatrics, Gynecology, and Obstetrics, Geneva University Hospitals, University of Geneva, 1205 Geneva, Switzerland

**Keywords:** biliary atresia, liver nodules, hepatocellular carcinoma, regenerative nodules, focal nodular hyperplasia

## Abstract

(1) Background: In patients with biliary atresia (BA) liver nodules can be identified either by pre-transplant imaging or on the explant. This study aimed to (i) analyze the histopathology of liver nodules, and (ii) to correlate histopathology with pretransplant radiological features. (2) Methods: Retrospective analysis of liver nodules in explants of BA patients transplanted in our center (2000–2021). Correlations with pretransplant radiological characteristics, patient age at liver transplantation (LT), time from Kasai hepatoportoenterostomy (KPE) to LT, age at KPE and draining KPE. (3) Results: Of the 63 BA-patients included in the analysis, 27/63 (43%) had nodules on explants. A majority were benign macroregenerative nodules. Premalignant (low-grade and high-grade dysplastic) and malignant (hepatocellular carcinoma) nodules were identified in 6/63 and 2/63 patients, respectively. On pretransplant imaging, only 13/63 (21%) patients had liver nodules, none meeting radiological criteria for malignancy. The occurrence of liver nodules correlated with patient age at LT (*p* < 0.001), time KPE-LT (*p* < 0.001) and draining KPE (*p* = 0.006). (4) Conclusion: In BA patients, pretransplant imaging did not correlate with the presence of liver nodules in explants. Liver nodules were frequent in explanted livers, whereby 25% of explants harboured malignant/pre-malignant nodules, emphasizing the need for careful surveillance in BA children whose clinical course may require LT.

## 1. Introduction

Biliary atresia (BA) is the main indication for liver transplantation (LT) in children [1,2]. Every BA patient, after Kasai hepatoportoenterostomy (KPE) or in the absence of it, will eventually develop some degree of liver fibrosis or cirrhosis. As biliary cirrhosis is associated with malignant transformation, children with BA warrant careful monitoring [3]. The usual modalities for the follow up of BA patients are ultrasound (US) and/or computed tomography (CT) and/or magnetic resonance imaging (MRI). If liver nodules identified on imaging exhibit malignant characteristics, biopsy is warranted [4]. The detection of malignant or premalignant nodules is clinically important, as it accelerates the need for LT [5]. It is known that explants of patients undergoing LT for BA can harbor various benign (regenerative nodules, focal nodular hyperplasia (FNH), adenomas), premalignant (low-grade and high-grade dysplastic nodules), or malignant (hepatocellular carcinoma (HCC), hepatoblastoma, and cholangiocarcinoma) nodules, yet the detection of these nodules through conventional imaging remains a challenge [3,4].

We aimed to analyze the histopathology of nodules identified on liver explants of patients undergoing LT for BA and to correlate histological findings with pre-LT radiological features. We hypothesized that there would be limited correlation between pre-LT radiological and post-LT pathological findings.

## 2. Materials and Methods

### 2.1. Patients

We conducted a retrospective study of all children diagnosed with BA and having undergone LT between 2000–2021 in our national referral center. Patient inclusion criteria were: primary diagnosis of BA and LT. Patient exclusion criteria were patients who did not have a primary diagnosis of BA, or BA patients who were not transplanted yet.

The following data were collected from the national BA database: demographics, age at KPE, cholangitis episodes (defined as i) fever associated with discolored stool and/or jaundice, or ii) fever associated with inflammatory parameters and/or cholestasis and/or increased transaminases and/or positive blood cultures), draining KPE (defined as conjugated bilirubin < 20 µmol/L at 6 months after KPE), pre-LT laboratory values (aspartate aminotransferase (AST), alanine aminotransferase (ALT), γ-glutamyl transferase (GGT), total and conjugated bilirubin, alpha-fetoprotein (AFP)), presence or not of pre-LT portal hypertension (defined as both splenomegaly + 2SD and thrombocytopenia (platelet count less than 100,000 G/l) or history of a complication of portal hypertension such as varices, ascites, etc.), age at LT, imaging (US performed at 6 weeks, 6 months, 1 year, 2 years pre-LT and/or CT performed prior to LT, see Section 2.2) and pathology results (see Section 2.3). The study was approved by the local ethics committee (CE 06-050).

### 2.2. Imaging Analysis

The routine imaging work-up consisted of color Doppler US and contrast enhanced CT scans that were performed by pediatric radiologists (3–25 years of experiece). The US machines were Acuson Sequoia 512 and Acuson S3000 (Siemens Healthineers, Erlangen, Germany) with curved-array 6 MHz and linear-array 9 MHz probes.

All CTs were obtained by using multi-detector machines: GE Lightspeed 16, GE Lightspeed VCT 64 (GE Medical Systems, WI, USA) and Somatom Definition Edge machine (Siemens Healthcare Systems, Erlangen, Germany). We used a low-dose technique based on patient weight and automatic exposure control. Parameters were: KV 80–120, 50–150 mA (care-dose modulation) and 2–5 mm slice thickness. Arterial and portal venous phase images were obtained after injecting Iohexol 300 (2 mL/kg). Imaging studies were reviewed by one author (MA) (25 years experience), first blindly, i.e., before knowing the results of the pathology report, and secondly after having received the results, to check for possibly missed nodules. For each nodule, the location (liver segments) and size (largest diameter), echogenicity on US and vascular uptake pattern on CT were reported.

### 2.3. Morphological and Histological Evaluation

Liver explants were submitted for gross and histological analysis. Specimens were handled according to guidelines, weighed and measured in all three dimensions, and serially cut along the transverse plane. For cirrhosis, the uniformity or variability of the nodules was recorded. After adequate fixation in 10% neutral buffered formalin, representative sections of the hilum, and of the right and left liver lobes were taken. Nodules standing out from the background liver because of size, differences in color or texture, or with a more pronounced bulging surface, were recorded and submitted for histological analysis.

Hematoxylin and Eosin stains were performed on 3 μm thick sections of the paraffin-embedded tissue. Special stains were routinely performed on selected paraffin blocks: Masson’s trichrome for collagen, reticulin for architecture evaluation, Perl’s Prussian blue for iron deposition, and PAS-diastase for hyaline globules. A reticulin stain and a Masson’s trichrome stain were performed on the nodules when required.

### 2.4. Statistical Analysis

Categorical variables were compared using the chi-square and Fisher exact test, accordingly. Continuous data were expressed as median and interquartile range. Continuous variables were compared using the Student *t*-test. Hazard ratios were estimated with the univariate Cox proportional hazards model. Survival curves were compared using the log-rank test. Differences were considered statistically significant if *p* < 0.05.

## 3. Results

During the study period, 168 patients had a LT. Sixty-three patients (33 females) were included in the study with a median age at LT of 12 (9–24.5) months. Sixty (60/63) of the patients underwent KPE. Median age at KPE was 58 (45–74) days. At 6 months, 27/60 (45%) patients had a draining KPE. Thirty-nine 39/60 (65%) patients after KPE experienced one or more cholangitis episodes and were treated with two to three weeks of IV antibiotics. Portal hypertension was present in 55/63 (87%) patients at LT. The median time from KPE to LT was 11 (6–73.5) months. Thirteen 13/63 (21%) patients had nodules on pretransplant imaging, while 27/63 (43%) had nodules on explants.

### 3.1. Nodules on Imaging

Thirteen 13/63 (21%) patients were radiologically diagnosed with 25 liver nodules prior to LT, none with features clearly in favor of malignancy (Table 1).

**Figure 1:**
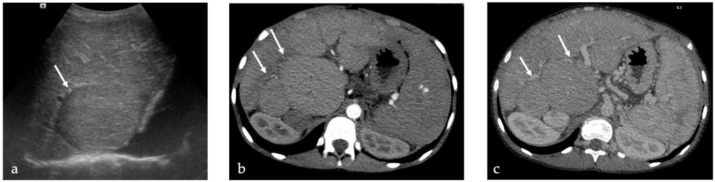
Macroregenerative nodules in a 14-years old boy with biliary atresia. US (**a**) with large isoechoic nodules with no vascularization on Doppler. CT with arterial (**b**) and venous (**c**) acquisition: multiple diffuse isodense iso-enhancing nodules.

Radiological findings in 7/13 (54%) patients correlated with the final histological diagnosis: one patient with FNH and 6 with regenerative nodules (Figure 1).

### 3.2. Nodules in Liver Explants

**Figure 2:**
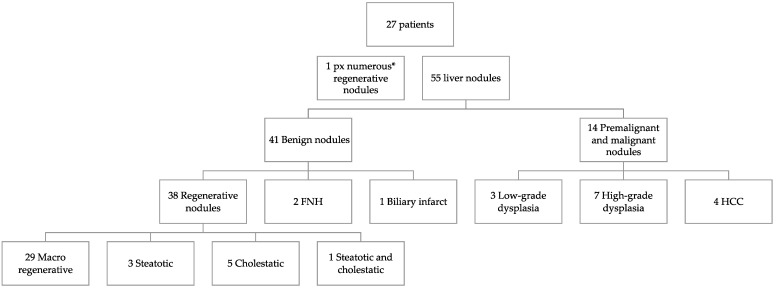
*Nodule types* on liver explants of biliary atresia patients (FNH, focal nodular hyperplasia; HCC, hepatocellular carcinoma; px, patient; * too-numerous-to-count).

**Figure 3:**
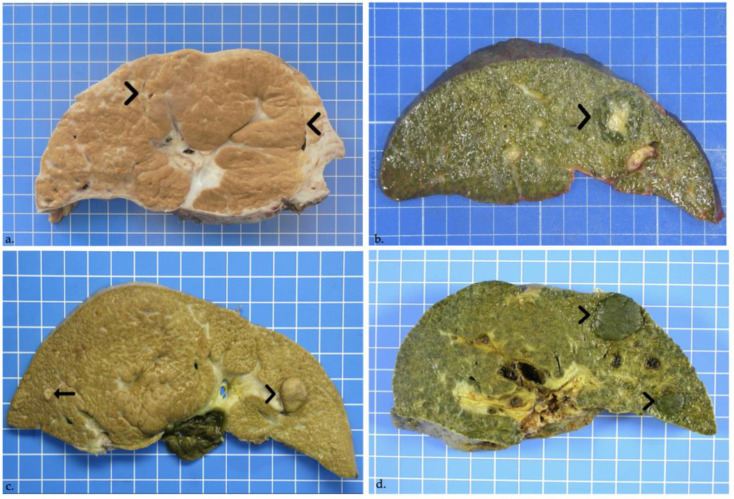
(**a**) Regenerative nodule in a male patient 7-years-old at liver transplantation. On cut section, a rather ill-defined 8-cm large regenerative macronodule involves segments IV, V and VIII (delineated by arrowheads). (**b**) Focal nodular hyperplasia in a 13-month-old girl. On cut section, a 2.5-cm lobulated and well-defined though unencapsulated cholestatic lesion with a central scar is seen in segment IV (arrowhead). (**c**) High-grade dysplastic nodule in a female patient aged 3 years and 5 months at transplantation. Macroscopy shows a 1.5-cm bulging brown nodule in liver segment III (arrowhead), and a further 0.4-cm nodule in segment VI (arrow). (**d**) Well-differentiated hepatocellular carcinoma. Two large cholestatic nodules in segments II and III, measuring 2.3 and 1.1 cm in greatest diameters, bulge out from the cut section (arrowheads).

**Figure 4:**
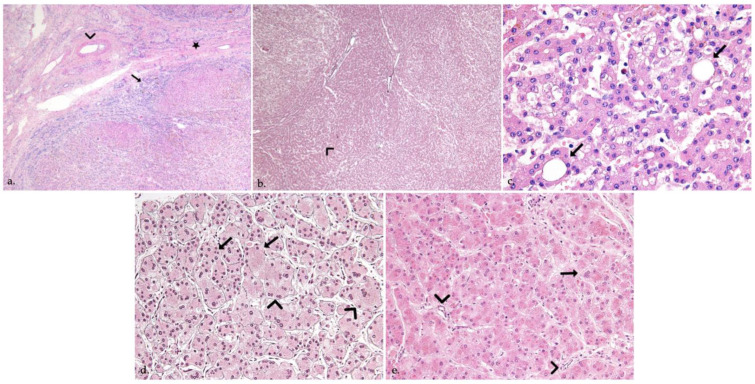
(**a**) Focal nodular hyperplasia in a 13-month-old girl. Histology shows benign hepatocellular nodules separated by thick fibrous septa (star) radiating from the central scar, containing thick-walled abnormal-appearing arteries (arrowhead) and a ductular reaction (arrow) (Hematoxylin & Eosin, H&E, original magnification ×40). (**b**,**c**) High-grade dysplastic nodule in a female patient aged 3 years and 5 months at transplantation, reticulin stain (5b, ×40) highlights a vaguely “nodular within nodule” growth (arrowhead), and increased cell density, while H&E stain (5c, ×400) shows small cell changes (arrowhead) and pseudoglands (arrows). (**d**,**e**) Well-differentiated hepatocellular carcinoma in a 1-year-old girl. Plate thickening (arrowheads) and focal loss of reticulin staining (arrows) is seen, together with variation in tumor cell size, multinucleation (arrow) and several unpaired arteries (arrowheads) (5d, reticulin stain, 5e, H&E, ×400).

Histological examination of liver explants revealed 55 nodules in 26 patients, 1 patient had 3 types of nodules and 4 other patients had 2 types of nodules. The liver of the 27th patient displayed nodules too-numerous-to-count (Figure 2). Half of the liver explants (14/27) presented with more than one nodule. Figure 1, Figure 3 and Figure 4 show representative imaging, gross and histological findings, in selected patients.

Most nodules were benign, with the majority being regenerative nodules (38) and the multiple nodules observed in the 27th liver explant, whereby 9 regenerative nodules displayed more pronounced cholestasis and/or steatosis than the background liver (Figure 3a). Identified in the majority of the patients presenting nodules (22/27), macroregenerative nodules were mainly seen in a central location, in segments IV, V and VIII (Table 2). Benign lesions also comprised 2 FNH, both located in segment IV (Figure 3b and Figure 4a), and one biliary infarct.

Ten premalignant and four malignant tumors were seen in 7 patients (7/27, 26%). Premalignant lesions were composed of 3 low-grade dysplastic nodules, and of 7 high-grade dysplastic nodules, measuring between 0.3 and 3.2 cm in the largest diameter (Figure 3c and Figure 4b,c). Malignant tumors were 4 well-differentiated HCCs, seen in 2 patients (2/27), measuring between 0.7 and 2.3 cm in the largest diameter (Table 2) (Figure 3d and Figure 4d,e). In the first patient, premalignant and malignant tumors co-existed: the liver explant of this female patient, aged 15 years at LT, showed co-occurrence of 2 well-differentiated HCCs, 2 high-grade dysplastic nodules and 3 macroregenerative nodules. The liver of the second 1-year-old patient with 2 well-differentiated HCCs, also showed a cholestatic 0.7 cm regenerative nodule.

### 3.3. Correlation between Imaging and Histopathology

Even after reviewing the US and CT scans, none of the malignant and premalignant nodules were detected on the pre-LT imaging. No further benign nodules were identified neither.

### 3.4. Patient Characteristics of Groups with and without Nodules in Liver Explants

**Figure 5:**
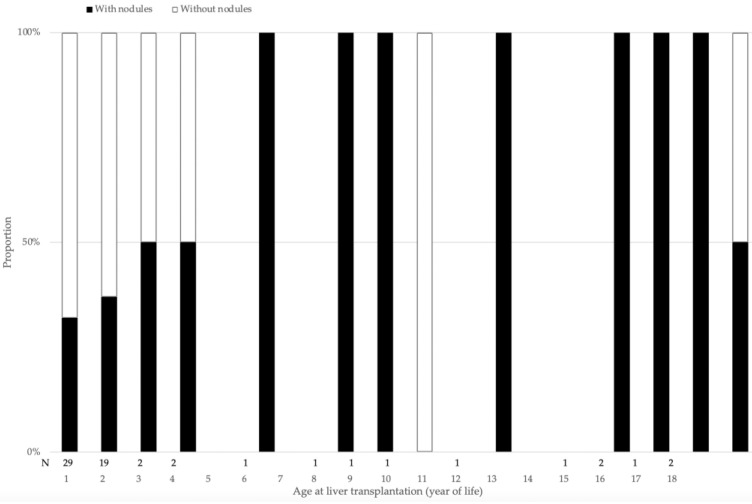
Percentage of patients *with* histologically detected nodules on their liver explant according to age.

Patients with histologically detected nodules on their liver explant were significantly older both at KPE (*p* = 0.05) and LT (*p* < 0.001) than patients without nodules. When LT was performed beyond the first year of life, significantly more explants presented *with* nodules, compared with patients receiving their LT in the first year of life: HR 1.6 [0.9–2.9] (*p* = 0.08); when LT was performed after the second year of life almost all liver explants displayed nodules (Figure 5). In the group *with* nodules, more patients had a draining KPE at 6 months, when compared with the group *without* liver nodules (*p* = 0.006). Significantly more patients had normal bilirubin values in their second year of life in the group *with* nodules when compared to the group *without* liver nodules (*p* = 0.003). No difference was identified between the two groups regarding the incidence of cholangitis (*p* = 0.63) or portal hypertension (*p* = 0.67) (Table 3). AFP was increased only in one of the two HCC patients.

Overall patient survival was not different in the group *with* nodules when compared with the group *without* liver nodules (*p* = 0.79). Likewise, there was no difference in survival between patients with premalignant or malignant nodules (*p* = 0.41). None of the patients had died or had been treated with chemotherapy at the end of follow-up (101 (48.5–156.5) months). No patient with malignant or premalignant nodules on liver explants presented with a recurrence.

## 4. Discussion

Nearly 50% of patients in this representative series of patients having undergone LT for BA displayed liver nodules upon histological examination of the explant, with one in four patients harboring malignant or premalignant lesions, most of which were not detected by pre-transplant imaging or serum AFP levels. Time from KPE to LT was associated with the occurrence of nodules, with more patients having identified nodules when LT was performed beyond the first year of life. The prevalence of liver nodules, their histopathological features, and the lack of correlation with imaging are all findings which differ somewhat from previous reports and warrant discussion.

### 4.1. Half of Explants with Nodules

The prevalence of liver nodules in this series reached nearly 50%. This is clearly higher than in previous series that report 11% of “liver nodules” on BA liver explants or [6] “benign and malignant” hepatic tumors in 8% of BA patients [3]. We hypothesized that the actual incidence of nodules in BA patients is underestimated due to underreporting.

There are several hypotheses concerning the physiopathology leading to these liver nodules. Vascular changes are frequently noted in the setting of chronic hepatic disease and also for BA patients we often observe an enlarged hepatic artery and a hypoplastic portal vein. These hemodynamic changes may induce the development of nodules such as FNH and regenerative nodules. Besides smoother bile drainage after KPE, Ijiri et al. formulate the hypothesis of a better blood supply of the porta hepatis, partially explaining the formation of hilar nodules [7]. Concerning the more peripheral nodules, Itai et al. formulate the hypothesis that the portal blood supply might not be able to reach the liver capsule in cases of diminished portal perfusion and thus contribute to the development of more peripheral nodules [7,8]. The link between KPE and nodules has been postulated by Hussein et al. in a comparative study of unoperated and post-KPE BA patients undergoing LT, no regenerative nodules were documented in the liver explants with diffuse biliary cirrhosis of the 6 patients without prior KPE. The authors therefore conclude that regenerative nodules are not purely a consequence of BA, and instead represent a consequence of KPE [9]. The highly regenerative modifications in the hilum might be a trigger for dysplasia and later HCC [9,10,11]. Further, the contact of the biliary epithelium with enteric contents has been reported as another accepted trigger for the development of bile duct malignancies [11,12]. Cholangitis might be another potential explanation to the appearance of liver nodules in BA patients. In our study, cholangitis occurrence was not statistically different between patients with and without nodules. Last, but not least, fibrosis grade at the time of KPE could play a role in the development of nodules. It has already been shown by Salzedas-Neto et al., that there is a negative correlation between the fibrosis grade on liver biopsy at KPE and a draining KPE [13]. As patients with a draining KPE exhibited more nodules in our series, we can speculate that there is a negative correlation between fibrosis grade and nodule occurrence.

### 4.2. Features of Benign Nodules

#### 4.2.1. Focal Nodular Hyperplasia

In our series, there were two FNHs, one detected by imaging. Both were centrally located, in segment IV. FNH, a benign hepatic lesion commonly seen in vascular liver disease rather than biliary cirrhosis, was found to be the most common lesion in a recent imaging series (6/13) [3]. There is one report of an FNH increasing in size after treatment of varices, congruent with the theory of hemodynamic changes [14]. Even if in 65% of the previously reported cases FNH are radiologically diagnosed, 3/17 were not detected on pre-LT imaging and other 3/17 were potentially diagnosed as HCC or hepatoblastoma [3,6,14,15,16,17]. In the present series, both of the FNH nodules were associated with other nodule types: a low-grade dysplastic nodule in a first patient and a macroregenerative nodule in a second one. The speculation that the hemodynamic changes encountered in the evolution of BA patients ultimately lead to the development of nodules needs further research [7].

#### 4.2.2. Regenerative Nodules

In this series, 24% of explanted livers displayed at least one regenerative nodule. Yet, the prevalence of regenerative nodules on explants of BA patients is described to be as low as 3.3% [4,7,18]. We assume that the discrepancy with previous reports may be due to underreporting. Indeed, only 22 cases of regenerative nodules are described in literature, versus 32 HCC [4,7,18], a ratio which seems very unlikely. Given that the malignant potential of macroregenerative nodules is still debated, close follow up of these patients is probably indicated [18,19].

For both FNH and regenerative nodules, the time elapsed from KPE to nodule detection is controversial: while it was found to be one per year in a cohort of 55 patients, other studies suggest a five to nine year-period until their first diagnosis [6]. Overall, in our series, patients with liver nodules had a longer time period from KPE to LT than those without nodules, suggesting that the longer the patient lives with his native liver, the higher the risk for developing nodules. That said, in our cohort clearly more patients develop nodules after the first year of life, underlining the need for screening already beyond the age of one year.

#### 4.2.3. Other Benign Nodules

Other benign nodules described in association with BA include mesenchymal hamartoma and adenomas, none of which were observed in our series [3,6,20]. Mesenchymal hamartoma and adenoma have both been presented as case reports in patients with BA. The pathophysiological relationship with BA is difficult to explain in both cases, raising the question of incidental findings [6,20].

### 4.3. Features of Malignant Nodules

The striking feature of the present series is the lack of correlation between pre-LT imaging and histopathological findings. None of the HCC nodules reported here were detectable on imaging, even on a post-hoc analysis, despite their detectable size (Table 2).

#### 4.3.1. Hepatocellular Carcinoma

The discrepancy between imaging and histopathology in the setting of BA has been reported in 32 pediatric cases [2,3,10,21,22,23,24,25,26,27,28,29,30,31,32,33]. The prevalence of HCC in explants of 544 children transplanted for BA was reported to be 1.2% [3,10,26]. Half of HCC seem to be missed during pre-LT imaging, and 41% of BA patients with HCC have a normal AFP [3]. The calculated sensitivity for the radiological diagnosis of HCC within the available literature of BA is 62.5% [2,3,10,21,22,23,24,25,26,27,29,30,31,32,33]. AFP was also of very limited use in our series, since it was only increased in one of the two patients with HCC. Indeed, AFP monitoring in BA patients and imaging has a low sensitivity of detecting small HCC. Nevertheless, increasing AFP in a BA patient should clearly encourage further imaging such as MRI with a hepatobiliary phase to rule out malignancy. The sensitivity of MRI to detect nodules is higher than both US and CT, and affords the opportunity to distinguish between regenerative nodules and malignancy using diffusion sequences and hepatospecific contrast agents. Small and borderline nodules (dysplastic and early HCC) may still be challenging to diagnose by MRI. Contrast-enhanced ultrasound, a non ionizing technique, is increasingly used in children for detecting and characterizing focal liver lesions and may be helpful in the follow-up of BA patients. As for other techniques, the difficulty lies in the analysis of nodules within a cirrhotic liver with global parenchymal changes [34]. Thus, in case of increased AFP and detected nodules, targeted biopsies should help to plan the appropriate management [28].

#### 4.3.2. Dysplastic Nodules

Dysplastic nodules are considered to be preneoplastic conditions [21]. Even if radiological investigations were negative for malignancy, a 3.3% incidence of HCC was reported in a series of dysplastic nodules [35]. The mean diameter of dysplastic nodules in our series correlated with the degree of dysplasia: the size increased from low grade dysplasia, to high grade dysplasia, to HCC, supporting the hypothesis of progression through the stages of carcinogenesis with the increasing diameter [5]. Given that half of the dysplastic nodules in the present series were located in the hilar area, especially segment IV, and the hilar region being known for having particular regenerative properties in BA patients, it is tempting to speculate about the role of the hilar hepatic regeneration in the development of dysplastic nodules and later HCC [9,10].

#### 4.3.3. Other Malignant Tumors

Other malignant tumors such as cholangiocarcinoma and hepatoblastoma have been reported to be found in liver explants of BA patients [2,36,37]. None of these tumors were found on liver explants in our series.

### 4.4. Limitations of This Study

The findings of this study have to be seen in the light of several limitations. First, there is a certain selection bias. This study started with a design in which we only investigated patients who finally underwent LT. KPE-succeeded BA patients spend a longer period of life with cirrhotic liver than KPE-failed patients. Therefore, KPE-succeeded BA patients might have more chances for the development of hepatic nodules. Associating successful KPE with the frequent occurrence of hepatic nodules after collecting LT candidates thus corresponds to a selection bias. However, this selection bias is inherent to the design and aim of our study that sought the correlations between pre-LT radiology and pathology of nodules of the explant.

Second, there is a literature gap regarding the pathophysiology of the development of nodules in BA patients. Our study incites for further research to clarify this aspect.

Third, the small sample size might limit the generalization of our results. Nevertheless, our data is representative as our center is the national referral center for BA patients and all patients are centralized.

## 5. Conclusions

Liver nodules were more frequently encountered in explanted livers after KPE than previously reported. A high proportion of liver nodules was not detected radiologically. One quarter of the lesions was malignant or pre-malignant, emphasizing the need for careful surveillance of BA patients and meticulous explant analysis. Older age at KPE, a draining KPE, and thus a longer time interval from KPE to LT, were associated with the presence of nodules on explants. How to improve detection of these nodules and whether patients require tailored follow-up are questions for future research.

## Figures and Tables

**Table 1:** Clinical, biological and imaging characteristics of patients with nodules on pretransplant *imaging*. LT, liver transplantation; US, ultrasound; CT, computed tomography; KPE, Kasai hepatoportoenterostomy; F, female; M, male; m, months; NA, not acquired.

Patient	Age at LT (m)	Time KPE-LT (m)	Sex	Size (mm)	Location	US	CT Arterial/Portal Phases	AFP (µg/L)	Pathology
1	6	4	F	17 25	IV	Hypoechoic Hypoechoic with central scar	Isodense/hypodense Isodense/hypodense with central scar	363	Macroregenerative nodule Focal nodular hyperplasia
2	10	8	F	5	V	No nodule	Hyperdense/hyperdense	3255	No nodule
3	10	8	M	6	VI	No	NA/Hyperdense	12.9	No nodule
4	13	11	M	7	IV	4 Hyperechoic hilar nodules	Hypodense/hypodense segment IV Hypodense/hypodense segment III	26.6	Regenerative steatotic nodule
5	15	14	M	11	VI	Hyperechoic nodule	Isodense/hypodense	7	Regenerative steatotic nodule
6	25	23	F	12	Left lobe	2 isoechoic nodules	No nodule	1.6	No nodule
7	47	6	M	60	III	Heterogeneous isoechoic	Isodense/isodense with a small central componenet hyperdense/hyperdense	32.7	Macroregenerative nodule
8	66	65	F	30	V	Isoechoic	NA	NA	Macroregenerative nodule
9	84	83	F	30 60	I V	No nodule	Isodense/isodense Isodense/hyperdense	2.1	Macroregenerative nodule
10	133	132	F	30	IV, VII, VIII	No nodule	Isodense/hypodense	1	Low-grade dysplastic nodule Regenerative cholestatic nodule
11	174	173	M	80	Diffuse	Isoechoic	Isodense/isodense	2.6	Macroregenerative nodules
12	201	200	F	27	III	No nodule	Isodense/isodense	2.3	High-grade dysplastic nodule
13	203	201	M	16	III	Hypoechoic nodule	No nodule	2.2	No nodule

**Table 2:** Histo-pathological characteristics of nodules identified in liver explants. * One nodule can overlap multiple liver segments. ** Largest diameter for every nodule, median (range) diameter for every *nodule type*. KPE, Kasai hepatoportoenterostomy; LT, liver transplantation.

Nodules	Nodule Type	Number of Nodules	Location * (Segments)	Size ** (mm)
**Benign nodules**	Macro regenerative nodules	29/55 (53%)	3xI/1xII/1xIII/8xIV/6xV/2xVI/ 5xVII/7xVIII	9 (3–80)
	Regenerative cholestatic nodules	5/55 (9%)	1xII/2xIII/1xVI/1xVII	12 (6–60)
	Regenerative steatotic nodules	3/55 (5%)	1xIII/1xIV	8.5 (5–12)
	Regenerative cholestatic and steatotic nodules	1/55 (2%)	1xVIII	7
	Focal nodular hyperplasia	2/55 (4%)	2xIV	20 (15–25)
	Biliary infarction nodules	1/55 (2%)	III	17
**Premalignant and malignant nodules**	Low-grade dysplasia nodules	3/55 (5%)	2xIV/1xV	7 (7–15)
	High-grade dysplasia nodules	7/55 (13%)	1xII 2xIII/1xIV/ 2xV/1xVI	7 (3–32)
	Hepatocellular carcinoma	4/55 (7%)	1xII/2xIII/1xV	14.5 (7–23)

**Table 3:** Characteristics of biliary atresia patients *with* and *without* nodules on liver explant. KPE, Kasai hepatoportoenterostomy; LT, liver transplantation.

Characteristics	With Nodules (*n* = 27)	Without Nodules (*n* = 36)	*p*-Value
Age at LT (months)	15 (5–207)	11 (3–203)	<0.001
KPE before LT	26/27 (96.2%)	34/36 (94.4%)	0.78
Age at Kasai (days)	53 (18–87)	64 (25–126)	0.05
Draining KPE at 6 months post KPE	17/26 (65.3%)	10/34 (29.4%)	0.006
Cholangitis episode(s) before LT	16/26 (61.5%)	23/34 (67.6%)	0.63
Portal hypertension	23/27 (85.1%)	32/36 (88.8%)	0.67
Direct bilirubin before LT (µmol/L)	34 (13–169.5)	163 (40–271.5)	0.39
Time period KPE to LT (months)	17 (3–1412)	8 (1–1338)	<0.001

## Data Availability

The data presented in this study are available on request from the corresponding author. The data are not publicly available due to ethical and legal reasons.
